# Whole-exome sequencing identified mutational profiles of urothelial carcinoma post kidney transplantation

**DOI:** 10.1186/s12967-022-03522-4

**Published:** 2022-07-21

**Authors:** Lee-Moay Lim, Wen-Yu Chung, Daw-Yang Hwang, Chih-Chuan Yu, Hung-Lung Ke, Peir-In Liang, Ting-Wei Lin, Siao Muk Cheng, A-Mei Huang, Hung-Tien Kuo

**Affiliations:** 1 Graduate Institute of Clinical Medicine, College of Medicine, Kaohsiung Medical University, Kaohsiung, Taiwan; 2 Division of Nephrology, Department of Internal Medicine, Kaohsiung Medical University Hospital, Kaohsiung Medical University, Kaohsiung, Taiwan; 3 School of Medicine, College of Medicine, Kaohsiung Medical University, Kaohsiung, Taiwan; 4 Department of Computer Science and Information Engineering, National Kaohsiung University of Science and Technology, Kaohsiung, Taiwan; 5 National Institute of Cancer Research, National Health Research Institute, Tainan, Taiwan; 6 Department of Urology, Kaohsiung Medical University Hospital, Kaohsiung Medical University, Kaohsiung, Taiwan; 7 Department of Urology, School of Medicine, College of Medicine, Kaohsiung Medical University, Kaohsiung, Taiwan; 8 Department of Urology, Kaohsiung Municipal Ta-Tung Hospital, Kaohsiung, Taiwan; 9 Graduate Institute of Medicine, College of Medicine, Kaohsiung Medical University, Kaohsiung, Taiwan; 10 Department of Pathology, Kaohsiung Medical University Hospital, Kaohsiung Medical University, Kaohsiung, Taiwan; 11 Department of Medical Research, Kaohsiung Medical University Hospital, Kaohsiung, Taiwan; 12 Ph.D. Program in Toxicology, College of Pharmacy, Kaohsiung Medical University, Kaohsiung, Taiwan; 13 Department of Biochemistry, School of Medicine, College of Medicine, Kaohsiung Medical University, Kaohsiung, Taiwan

**Keywords:** Kidney transplantation, Malignancy, Urothelial carcinoma, Whole exome sequencing, Mutations

## Abstract

Kidney transplantation is a lifesaving option for patients with end-stage kidney disease. In Taiwan, urothelial carcinoma (UC) is the most common de novo cancer after kidney transplantation (KT). UC has a greater degree of molecular heterogeneity than do other solid tumors. Few studies have explored genomic alterations in UC after KT. We performed whole-exome sequencing to compare the genetic alterations in UC developed after kidney transplantation (UCKT) and in UC in patients on hemodialysis (UCHD). After mapping and variant calling, 18,733 and 11,093 variants were identified in patients with UCKT and UCHD, respectively. We excluded known single-nucleotide polymorphisms (SNPs) and retained genes that were annotated in the Catalogue of Somatic Mutations in Cancer (COSMIC), in the Integrative Onco Genomic cancer mutations browser (IntOGen), and in the Cancer Genome Atlas (TCGA) database of genes associated with bladder cancer. A total of 14 UCKT-specific genes with SNPs identified in more than two patients were included in further analyses. The single-base substitution (SBS) profile and signatures showed a relative high T > A pattern compared to COMSIC UC mutations. Ingenuity pathway analysis was used to explore the connections among these genes. *GNAQ*, *IKZF1*, and *NTRK3* were identified as potentially involved in the signaling network of UCKT. The genetic analysis of posttransplant malignancies may elucidate a fundamental aspect of the molecular pathogenesis of UCKT.

**Supplementary Information:**

The online version contains supplementary material available at 10.1186/s12967-022-03522-4.

## Introduction

Kidney transplantation is a lifesaving option for patients with end-stage kidney disease (ESKD) because it significantly improves survival; it has a mortality rate that is 63–80% lower than that of continued dialysis [1, 2]. Modern immunosuppressive agents have tremendously reduced the incidence of acute rejection within the first year and achieved outstanding short-term patient and graft survival [3]. However, the long-term survival rate of patients who receive kidney transplants is low. Recipients of a kidney transplant experience various complications, including cardiovascular disease and posttransplant malignancies (PTMs), that lead to morbidity and mortality [3].

The incidence of cancer in recipients of a kidney transplant is at least 2 to 4 times higher than that of their age-matched and gender-matched counterparts in the general population [4–6]. Studies from Western countries have reported that nonmelanoma skin cancer is the most common PTM [7]. The incidence of skin cancer is lower in Asian countries. Urothelial carcinoma (UC) of the bladder and urinary tract is the most common PTM in Taiwan and Mainland China, comprising approximately 40% of PTMs [8, 9]. In a nationwide cohort population-based study, Tsai et al. reported that recipients of a heart, lung, kidney, or liver transplant exhibited a risk of de novo cancer that was three times higher than that of the general population [10]. The standardized incidence ratio of urinary tract malignancies (among which bladder cancer was the most common) among recipients of a kidney transplant was 10.93 (95% CI, 9.20–12.99) [10]. Furthermore, in our previous single-center retrospective study, the most common PTMs were UC and hepatocellular carcinoma [11].

UC has a high mutational burden and exhibits greater molecular heterogeneity than do other solid tumors [12]. Different genetic alterations and pathogenic pathways occur in UCs at various anatomical locations [13, 14]. Somatic genetic mutation is a highly influential factor in UC tumorigenesis and progression. An abundance of information regarding somatic alteration in UC has been published due to rapid progress in next-generation sequencing [15]. According to the Cancer Genome Atlas (TCGA) database, the genes with the most common mutations in bladder UC are *TP53*, *KDM6A*, and *ARID1A* [15, 16]. *TP53* mutations were commonly observed in high-grade tumors, whereas *FGFR3*, *CREBBP*, and *STAG2* mutations were more commonly observed in low-grade tumors [15].

Recipients of a kidney transplant are prone to developing cancer due to underlying diseases leading to renal failure, chronic infections by oncogenic viruses, immunocompromised status related to treatment with immunosuppressive drugs, nutritional deficiencies, or altered deoxyribonucleic acid (DNA) repair. Knowledge of the underlying somatic genomic alterations in UC developed after kidney transplantation (UCKT) is limited. To our knowledge, no unbiased systematic effort has been made to describe genomic alterations in UCKT.

Whole-exome sequencing (WES) has been extensively applied to identify drivers and somatic alterations in diseases, including cancer genome profiles, in both research and clinical settings [17, 18]. WES is an effective tool for medical genetic research because it targets almost all protein-coding regions in the human genome. In this study, we performed WES and analyzed UC tissues from recipients of a kidney transplant (the UCKT group) and patients on hemodialysis (the UCHD group) to further characterize the unique genomic landscape of UCKT.

## Materials and methods

### Sample collection and DNA extraction

The sample collection and study protocol were conducted under the Institutional Review Board of Kaohsiung Medical University Hospital (KMUHIRB-G(I)-20150030). Written informed consent was obtained from all the patients, and all clinical investigations were conducted in accordance with the Declaration of Helsinki.

A total of 12 tumor samples, comprising 7 formalin-fixed archival UC samples from 6 recipients of a kidney transplant and 5 UC samples from patients on hemodialysis, were collected. Recipients of a kidney transplant who had a diagnosis of UC before their respective transplants were excluded. Because chronic kidney disease (CKD) and ESKD are risk factors for UC, five patients on hemodialysis who received a diagnosis of UC after the commencement of dialysis were selected to form the control group. Each patient’s medical history was confirmed by chart review. None of the patients had previously received chemotherapy or radiotherapy.

After a pathologist reviewed all the samples, the tumor samples from the paraffin blocks were macrodissected. All the specimens were placed in Eppendorf containers to prevent tissue cross-contamination. Genomic DNA was extracted using a FavorPrep formalin-fixed paraffin-embedded (FFPE) Tissue DNA Extraction Micro Kit (Favorgen Biotech, Pingtung, Taiwan) from FFPE tumor tissue. The DNA was quantified and qualified using Qubit 4 Fluorometer (Thermo Fisher Scientific, Waltham, USA), 4150 TapeStation System (Agilent, Santa Clara, USA), and Nanodrop spectrophotometer (ASP-2680, Celltagen, Seoul, Republic of Korea) according to the manufacturer’s protocol. One microgram DNA from each tumor sample was subjected to run the WES analysis.

### Whole-exome sequencing

Library enrichment for WES was conducted using a SureSelect^XT^ V6_r2 reagent kit (Agilent Technologies, Santa Clara, CA, USA). The enriched samples were sequenced using an Illumina HiSeq2500 system (San Diego, CA, USA) with a 2 × 76–base pair (bp) paired-end sequencing approach. The mean coverage of the exomes was 79.76 × , and more than 96% of the exomes had a coverage higher than10 × . The sequencing data were annotated according to the GRCh37/hg19 reference genome.

The data were examined using a data analysis pipeline developed at the Cologne Center for Genomics (CCG) [19]. Initially, raw sequencing reads were mapped to human genome reference (hg19) using Burrows-Wheeler Alignment tool (BWA) [20] followed by duplication marking using Picard [21].

Subsequently, base quality score recalibration and local indel realignment were performed according to Genomic Analysis Toolkit (GATK) practice guideline [22, 23]. After the post-alignment improvements, variant calling was performed using GATK’s unified-genotyper and SAMtools’ mpileup [24]. The variant lists from both callers were merged and annotated using various public databases: dbSNP [25], 1000 Genomes Project [26], Exome Variant Server [27], HGDM professional database [28], dbVAR and DGVa [29], GERP, and Ensembl [30]. The functional effect of variants were predicted using PolyPhen [31], SIFT [32], and RVIS [33]. All of these downstream analysis has been assembled together with a few scripts developed in-house. Splice site analysis based on Yeo [34] et al. was performed. Finally, the annotated variant list is uploaded to CCG’s web interface and VARBANK database [35]. Scripts developed in-house at the Cologne Center for Genomics were applied to detect protein changes, affected donor and acceptor splice sites, and overlaps with known variants. Our analysis focused on single-nucleotide variants/polymorphisms (SNVs or SNPs) and insertions or deletions (InDels) that may result in alterations in primary protein structure or in strong splice site effects [34].

### Identifying specific genes in UCKT

The processed results of all 12 samples were then downloaded from VARBANK analysis platform. A series of steps were performed to identify cancer driver genes that contain novel mutations and are not reported previously in UCKT patients. First, mutations with dbSNPs annotations and with low allele frequencies (≤ 5%) were excluded from the data sets. Mutations at the same genomic location that were annotated as different transcripts were counted as one occurrence.

To ensure that these genetic alterations identified in our study were associated with tumor development, we identified genes with these mutations and using these gene names to search against the Catalogue of Somatic Mutations in Cancer (COSMIC) [36]. If in our list, a gene is also a reported cancer driver gene in COSMIC, the gene and its mutations was retained for further analysis. In other words, these newly identified mutations in our samples were considered potential driver mutations.

Subsequently, we utilized previous studies or databases that had analyzed bladder cancer including the COSMIC [36], the Integrative Onco Genomic (IntOGen) cancer mutations browser [37], the TCGA bladder tumor database [15], and the Chinese bladder cancer genome [38] to compare with our data. Cancer driver genes that were retained in the previous step but had not been reported in these bladder cancer studies were of great interest.

**Fig. 1:**
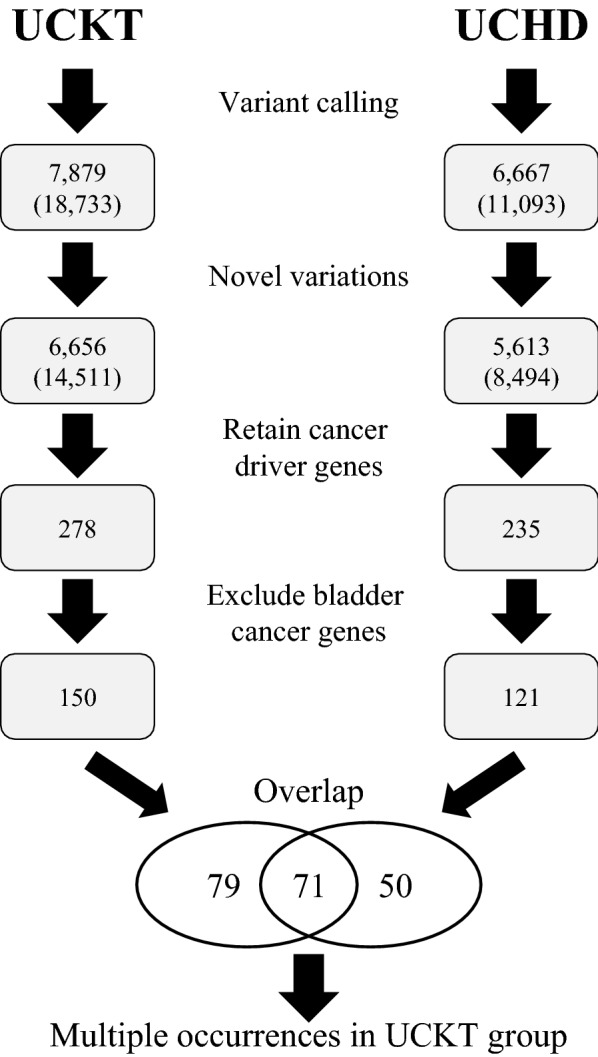
Flow chart of data analysis. The WES data from each patient is subjected to genome mapping and variant calling (Variant calling). Then the data was divided into 2 groups. UCKT; N = 7 and UCHD; N = 5. The mutations were then pooled together in each group and known dbSNPs were excluded (Novel variations). The COSMIC-annotated cancer driver genes were selected (Retain cancer driver genes), but next, bladder cancer-related genes were removed from the list (Exclude bladder cancer genes). Genes that were unique in each group were identified (Overlap). Final list of genes contains those occurred in two or more UCKT patients (Multiple occurrences in UCKT group). Numbers represent gene counts, except those in parentheses, which are nucleotide mutations

Then we selected the above driver genes which were identified in UCKT but not in UCHD groups. Finally, we reported genes that were found in two or more UCKT patients. Figure 1 illustrates the data processing steps and corresponding mutations and gene numbers for UCKT and UCHD groups. COSMIC UC single-base mutation data was retrieved from the Cancer Browser in COSMIC [39] by using the selection of Primary site (Urinary tract), Sub site (Bladder), Histology (Carcinoma) and Sub histology (Transitional cell carcinoma). The mutational signatures were conducted using the SigProfiler tools (MatrixGenerator, Extractor and Plotting) [40]. Data from UCKT, UCHD and COSMIC UC were applied separately.

### Sanger sequencing analysis for confirmation of somatic mutations

Genomic DNA from patients’ peripheral blood mononuclear cells (PBMC) was used as reference for detecting somatic mutations. We used a FavorPrep Blood / Cultured Cell Genomic DNA Extraction Mini Kit (Favorgen Biotech, Pingtung, Taiwan) to extract the genomic DNA from PBMC. All the somatic mutations of the selected genes were independently verified through Sanger sequencing of the PBMC genomic DNA. Primer pairs were designed using the University of California, Santa Cruz Genome Browser [41] and Primer-BLAST Browser of the National Institutes of Health [42].

The target regions were investigated using a touchdown polymerase chain reaction (PCR) protocol. The PCR mixture contained primers at 5 μmol L^−1^ each, Taq DNA polymerase (Vazyme Biotech, China), and 50 ng/uL genomic DNA. The following touchdown PCR protocol was executed in a T100 thermal cycler (Bio-Rad Laboratories, CA, USA): initial denaturation at 94 °C for 5 min; annealing temperature decreased by 0.5 °C every other cycle: 94 °C for 30 s, annealing from 72 to 55 °C for 30 s, 72 °C for 30 s; followed by a cycle of 94 °C for 30 s, 55 °C for 30 s, and 72 °C for 30 s; and extension at 72 °C for 10 min. The PCR products were resolved in 1.5% (w/v) agarose gel for 20 min at 100 V in 1 × TAE buffer and stained with Gel Red biosafety dye (Biotium, CA, USA). The gel was documented on a UV transilluminator (Major Science, Taiwan). The DNA sequencing was performed at the core facility of the National Yang Ming Chiao Tung University (NYCU) Genome Center.

### Analysis of gene expression profile by ingenuity pathway analysis

The Ingenuity Pathway Analysis (IPA version 68752261; QIAGEN) tool was used to establish protein networks. The main molecules detected from WES analysis and Sanger sequencing verified from the PBMC genomic DNA PCR products were set as the focusing molecules and were analyzed using the build tool to determine how their relationships were affected by UC. The molecules from the QIAGEN knowledge base were then added to the network according to their specific ontological associations.

### Copy number variation (CNV) detection

CNV analysis was performed using ExomeDepth (ver 1.1.15) R software package, which uses read depth data to call CNVs from exome sequencing experiments. We generated read count data from WES BAM files, and compare the count results with an aggregate reference made up of samples from the same sequencing run to determine copy number at exon level resolution. Data were analyzed according to the ExomeDepth standard procedure [43].

## Results

### Baseline characteristics

The baseline characteristics of all the patients and their clinical and pathological features are listed in Table 1. All patients were female and sporadic, with no family history of UC nor any history of smoking. The majority (10 out of 12) had high-grade tumors. The KT80 and KT721 samples were from the same patient, a woman who underwent surgical removal of different parts of her genitourinary tract system over 5 years (Table 1). In the KT recipients, all the tumors occurred in their native genitourinary systems. The patients underwent induction treatments with steroids and IL-2 receptor antagonists. Tacrolimus, mycophenolate mofetil, and steroids were selected as maintenance agents. The average age at UC diagnosis was younger in the UCKT group than in the UCHD group (55 vs. 61.6 years, respectively; Table 2).

**Table 1:** Baseline characteristics and clinic-pathologic features of all patients

Patient ID	Gender	Age at diagnosis	HBV/HCV	Chinese Herb history	Occupation	Kidney transplant	Induction	Maintenance	Acute rejection	Location	Tumor pathology	Treatment
KT17	F	48	No	Unknown	Bio-engineer	Yes		Tac + MMF + Pred	Yes	Bladder	Infiltrating UC high grade, pT3NoMx,	Partial cystectomy
KT79	F	61	No	Yes	Housewife	Yes		Tac + MMF + Pred	Yes	Bladder, ureter	Noninvasive UC, high grade, pTa	Left nephrectomy
#KT80	F	51	HCV	Unknown	Housewife	Yes*		Tac + MMF + Pred + MTOR	No	Ureter (Lt)	Infiltrating UC high grade, pT2Nx,	Bilateral nephrectomy
KT81	F	63	HBV	Unknown	Housewife	Yes*	Steroid + IL2RA	Tac + MMF + Pred + MTORi	No	Renal Pelvis	Infiltrating UC high grade, pT2Nx,	Left nephrectomy
KT720	F	46	No	Unknown	Medical	Yes		Tac + MMF + Pred + MTORi	No	Renal Pelvis	Infiltrating UC, high grade, pT3	Right nephrectomy
#KT721	F	55	No	Unknown	Housewife	Yes*		Tac + MMF + Pred + MTOR	No	Bladder	Non-invasive papillary UC, high grade, pTa	TURBT
KT722	F	61	No	Unknown	Housewife	Yes		Tac + MMF + Pred	No	Renal Pelvis	Infiltrating UC,high grade, pT2aNx	Left nephrectomy
KT73	F	52	No	Unknown	Housewife					Bladder, ureter	Non-invasive Papillary UC, low grade	Right nephrectomy and cystectomy
KT77	F	56	HCV	Yes	Housewife					Bladder	Infiltrating UC,high grade, pT1Nx	Radical cystectomy and bilateral nephrectomy
KT78	F	70	No	Unknown	Sales person	No	N/A	N/A	N/A	Bladder + ureter	Infiltrating UC, high grade, pT3aNx	Radical cystectomy
KT595	F	64	No	Yes	Worker					Renal Pelvis	Infiltrating UC, low grade, pT1NxMx	Left nephrectomy
KT596	F	66	No	Unknown	Housewife					Bladder	Non-invasive Papillary UC, high grade	Left nephrectomy

Abbreviations: *UC*, Urothelial carcinoma; *Tac*, Tacrolimus; *MMF*, Mycophenolate myfortil; *Pred*, prednisolone; *MTORi*, Mammalian target of rapamycin inhibitors. *N/A*: not applicable

*Transplant Tourism in Mainland China

#KT80 and KT 721 sample are from the same patient at different time of surgery

**Table 2:** Summary of whole-exome sequence analysis of human urothelial carcinomas in kidney transplantation (UCKT) and hemodialysis (UCHD)

		UCKT (N = 7)	UCHD (N = 5)
	Average age at diagnosis (years)	55	61.6
	Mean coverage	81	77
Sequencing results	Targeted bases with at least 10 reads (%)	96.19	95.78
	Known SNPs identified in targeted region^*^	4198	2574
Somatic mutation	Total somatic mutations *	18,703	11,060
	Insertion or deletion	271	188
	Single based substitution	18,432	10,872
	Synonymous	1976	65
	Missense	13,980	8782
	Nonsense	948	550
	Frameshift	288	186
	Splice acceptor	904	923
	Splice donor	301	331
	Nonstop	35	35
Frequency (%) **	C > A	7.03	6.72
	C > G	7.74	6.78
	C > T	27.69	23.96
	T > A	40.03	44.63
	T > C	11.80	12.06
	T > G	5.70	5.86

The WES results of the 12 samples were aligned to the human reference genome (hg19) and further subjected for bioinformatics analysis. UCKT; N = 7 and UCHD; N = 5

*The number was calculated as the summation of the samples in each group

**The number was calculated as the average of the samples in each group

### Whole-exome sequencing result analysis of UC

After WES was performed, the results were aligned to the human reference genome (hg19) and further used in a bioinformatics analysis. At least 100 million reads were collected from each WES sample. In the UCKT and UCHD groups, the mean coverages were 81 and 77 reads for UCKT and UCHD, respectively, and 96.18% and 95.78% of the coding exons, respectively, were covered by at least 10 reads (Table 2). A total of 18,733 and 11,093 variants were identified from the UCKT and UCHD cohorts (Fig. 1).

The majority of the mutations were unique to individual patients. Mutations were slightly more common in the UCKT group than in the UCHD group. An analysis of the WES data revealed an average of 564 known SNPs per individual. To identify new UC-related alterations, the mutations with dbSNP annotations were excluded.

We used the COSMIC cancer driver genes database to identify new SNPs in our cohorts. During this step, 278 and 235 genes in the UCKT and UCHD groups, respectively, were retained (Fig. 1). To identify oncodriver genes specific to our cohorts (i.e., previously unknown to be related to UC), we first collected UC-related studies from the COSMIC [36], IntOGen cancer mutations browser [37], TCGA bladder tumor database [15], and Chinese bladder cancer genome [38] and we excluded genes that had already been reported in these studies. Ultimately, we identified 150 and 121 genes in the UCKT and UCHD groups, respectively, that had never previously been known to be associated with UC. Of these cancer driver genes, 79 were uniquely identified in the UCKT cohort, and 17 of them (*BTK*, *CARD11*, *ELL*, *FNBP1*, *GNAQ*, *HOXD13*, *IKZF1*, *MAX*, *MLLT10*, *NTRK3*, *PAX5*, *SEPTIN6*, *SEPTIN9*, *SH3GL1*, *SLC34A2*, *TAL1*, and *TRAF7*) exhibited mutations that occurred in two or more patients (Table 3).

**Table 3:** Gene lists 17 genes uniquely identified in urothelial carcinomas post kidney transplantation

Symbol	Entrez gene name	NCBI reference sequence	Location	Family
*BTK*	Bruton tyrosine kinase	NM_000061	Cytoplasm	Kinase
*CARD11*	caspase recruitment domain family member 11	NM_032415	Cytoplasm	Kinase
*ELL*	Elongation factor for RNA polymerase II	NM_006532	Nucleus	Transcription regulator
*FNBP1*	formin binding protein 1	NM_015033	Nucleus	Enzyme
*GNAQ*	G protein subunit alpha q	NM_002072	Plasma membrane	Enzyme
*HOXD13*	Homeobox D13	NM_000523	Nucleus	Transcription regulator
*IKZF1*	IKAROS family zinc finger 1	NM_001220775	Nucleus	Transcription regulator
*MAX*	MYC associated factor X	NM_197957	Nucleus	Transcription regulator
*MLLT10*	MLLT10 histone lysine methyltransferase DOT1L cofactor	NM_001195626	Nucleus	Transcription regulator
*NTRK3*	Neurotrophic receptor tyrosine kinase 3	NM_001012338	Plasma membrane	Kinase
*PAX5*	Paired box 5	NM_016734	Nucleus	Transcription regulator
*SEPTIN6*	Septin 6	NM_145800	Cytoplasm	Other
*SEPTIN9*	Septin 9	NM_001113495	Cytoplasm	Enzyme
*SH3GL1*	SH3 domain containing GRB2 like 1, endophilin A2	NM_001199943	Cytoplasm	Other
*SLC34A2*	Solute carrier family 34 member 2	NM_006424	Plasma membrane	Transporter
*TAL1*	TAL bHLH transcription factor 1, erythroid differentiation factor	NM_003189	Nucleus	Transcription regulator
*TRAF7*	TNF receptor associated factor 7	NM_032271	Cytoplasm	Enzyme

Table 4 showed the related genes with non-synonymous mutations. After deleting the genes without amino acid change, only 14 genes (*CARD11*, *FNBP1*, *GNAQ*, *HOXD13*, *IKZF1*, *MAX*, *MLLT10*, *NTRK3*, *SEPTIN6*, *SEPTIN9*, *SH3GL1*, *SLC34A2*, *TAL1*, and *TRAF7*) were left.

**Table 4:** Genetic characteristics of urothelial carcinomas post kidney transplantation

Identified genes	Patient ID	Number of tumors with nonsynonymous mutation (frequency)	Chromosome	Position	Complementary DNA	Protein
*CARD11*	KT80			2,979,501	c.746A > T	p.Q249L
	KT80	3	7	2,959,246	c.2270G > T	p.G757V
	KT721			2,979,984	c.298G > A	p.E100K
*FNBP1*	KT720 KT721	2	9	94,012,949 132,686,175	c.1118A > T c.1279A > T	p.E373V p.R427*
*GNAQ*	KT80, KT721 KT79	3	9	80,537,229 80,537,135	c.169A > T c.263A > T	p.K57* p.Q88L
*HOXD13*	KT79, KT722	2	2	176,957,650	c.32G > C	p.G11A
*IKZF1*	KT80 KT81	2	7	50,450,403 50,367,232	c.326C > G c.41-2A > T	p.S109C p.?
*MAX*	KT79, KT81	2	14	65,472,892	c.*30delC	p.?
*MLLT10*	KT720 KT80, KT721 KT81	4	10	21,903,809 21,959,631 21,875,221	c.559A > T c.1049A > T c.241-2A > T	p.N187Y p.Q350L p.?
*NTRK3*	KT80, KT721 KT79	3	15	88,799,202 88,727,498	c.183 T > A c.281 T > A	p.D61E p.L94H
*SEPTIN6*	KT80, KT721 KT81	3	X	118,763,455 118,786,815	c.1106A > T c.528 + 2 T > A	p.D369V p.?
*SEPTIN9*	KT79, KT720	2	17	75,471,875	c.275A > T	p.E92V
*SH3GL1*	KT81 KT79	2	19	4,362,684 4,361,737	c.634C > T c.823G > C	p.R212W p.G275R
*SLC34A2*	KT80, KT721 KT17	3	4	25,674,846 25,677,770	c.1183G > A c.1469A > T	p.A395T p.H490L
*TAL1*	KT720 KT720, KT722 KT720	3	1	47,691,377 47,685,603 47,685,597	c.184G > C c.785 T > G c.791C > G	p.G62R p.V262G p.A264G
*TRAF7*	KT79 KT722	2	16	2,222,207 2,221,345	c.491A > T c.429C > T	p.D164V -

The 17 UCKT-unique genes were listed in Table 3. The chromosome location and the frequency and mutations of 14 selected genes were summarized in Table 4

To verify the tumorigenic nature of the 14 genes in the UCKT cohort (*CARD11*, *FNBP1*, *GNAQ*, *HOXD13*, *IKZF1*, *MAX*, *MLLT10*, *NTRK3*, *SEPTIN6*, *SEPTIN9*, *SH3GL1*, *SLC34A2*, *TAL1*, and *TRAF7*), we developed a reliable PCR system to validate the mutations in the patients’ PBMC genomic DNA and subjected to Sanger sequencing analysis. The results revealed that mutations in *GNAQ*, *SEPTIN 6*, *NTRK3*, and *IKZF1* occur only in tumor samples. The possible interactions among these genes deserved further pathway analysis.

### SBS and CNV analysis

**Fig. 2:**
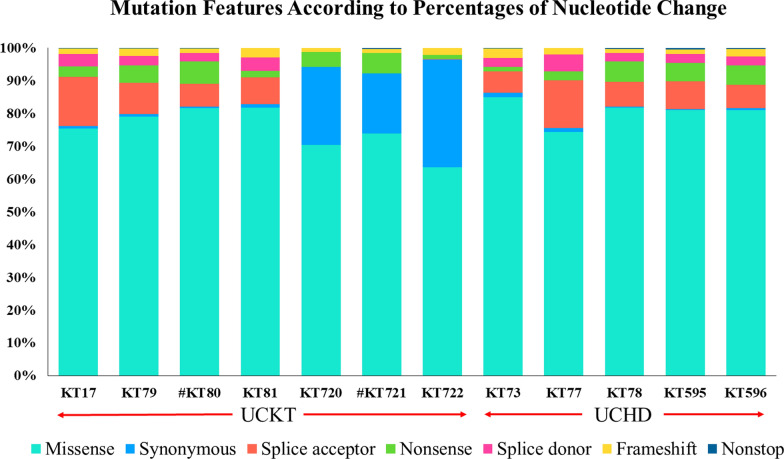
The mutational features of the individual UC patients. The mutational features of individual patients were plotted according to the percentages of nucleotide changes

The mutations were classified into two categories: single-base substitutions (SBSs) and InDels (insertions/deletions) (Table 2). The single-base substitution category was further divided into six subcategories: synonymous, missense, nonsense, frameshift, splice acceptor or donor, and nonstop (Table 2). The frameshift subcategory comprising one-base-pair differences between the alleles and reference sites, whereas the InDels category comprises mutations involving longer regions. Missense mutations were the most common type in both the UCKT and UCHD groups (74.75% and 79.40%, respectively; Table 2). These mutations are associated with amino acids changes in protein sequences, thereby affecting the structures and functions of the proteins that their respective genes encode. Interestingly, synonymous substitutions were much more common among the patients with UCKT than among those with UCHD (10.57% vs. 0.59%), which might indicate that the overall mutation rate increases after kidney transplantation, resulting in a higher risk of cancer. Among the nucleotide changes, T > A was the most common one in both the UCKT and UCHD groups (Table 2). In addition, in a TCGA study, 51% of all the mutations in bladder cancers were TpC > T or G mutations and 3.8% were TpC > A mutations [15]. Figure 2 illustrates the mutational features of the patients. Although missense mutations were the most common somatic mutations, followed by nonsense mutations, splice site mutations were also common. The effects of splice site mutations are difficult to determine; however, they may affect protein function by causing alternative splicing, intron retention, or exon skipping.

**Fig. 3:**
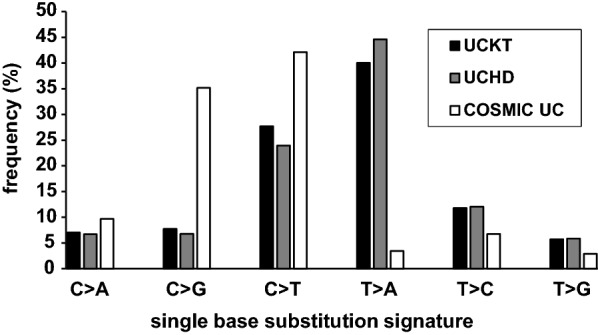
The comparison of single-base substitution (SBS) profile of datasets. The SBS data from COSMIC UC was retrieved from COMSIC Cancer browser. The six classes of SBS pattern from UCKT, UCHD and COSMIC UC were calculated and showed as the frequency of total events, respectively

To further analyze the mutation signatures and compare to other cohort, total of 95,657 unique mutations were obtained from the Cancer browser in COSMIC for UC. The distribution of six classes of SBS showed distinct patterns among UCKT, UCHD and COMIC UC data (Fig. 3). The UCKT and UCHD showed relative high percentage in T > A (40.03% and 44.63%) while COSMIC showed high C > T (42.12%) and C > G (35.17%) mutations.

The results from SigProfiler tool [44] showed that both SBSs in UCKT and UCHD presented highly similar to the SBS22 signature (83.6% and 81.12%) with the proposed aetiology “Aristolochic acid exposure”, SBS5 (10.44% and 11.18%) with “unknown aetiology mutational burden is increased in bladder cancer samples with ERCC2 mutations and in many cancer types due to tobacco smoking” and SBS1 (5.96% and 7.7%) with “an endogenous mutational process initiated by spontaneous or enzymatic deamination” while COSMIC UC is similar to SBS5 (27.12%) SBS1 (4.48%), SBS13 (40.36%) and SBS2 (28.04%), respectively (Additional file 1: Fig. S1). These findings might indicate the possible unique aetiology for the UC development in Taiwan.

In addition, CNV data was retrieved from the WES results and divided into two groups. We observed 1495 and 1033 events in UCKT and UCHD groups. Using the start site as a reference, events occur in two or more patients in either group were selected. We removed records that were found in both groups and only reported those that were specific in UCKT. We identified 208 events with 92 unique CNVs in UCKT group (Additional file 2: Table S1). None of 14 genes of interest in Table 4 were found in this CNV analysis.

### IPA network analysis

**Fig. 4:**
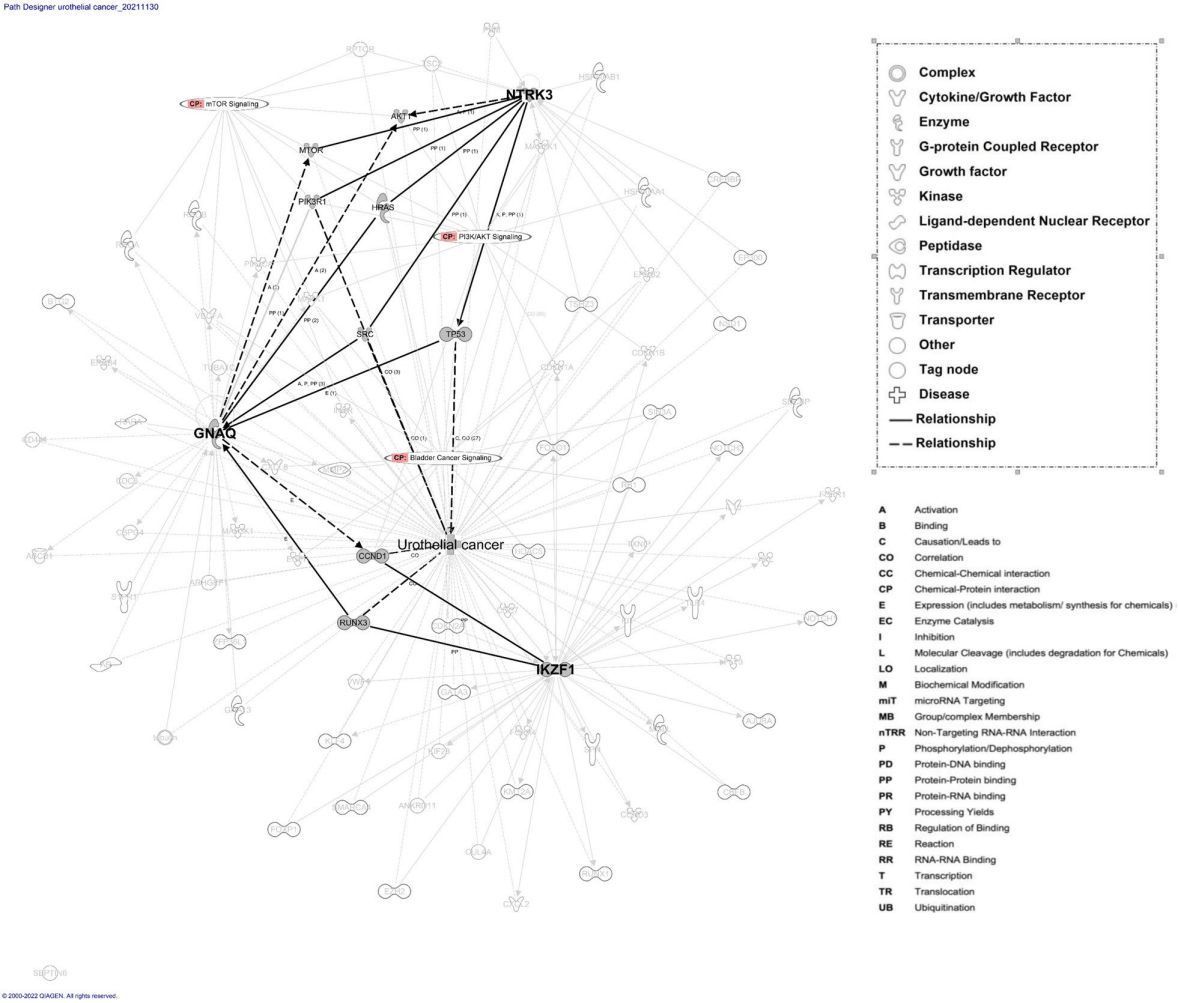
IPA based protein networks involved in target protein and disease. The focused molecules represented in grey color and bold were NTRK3, GNAQ and NTRK3 which related to Urothelial cancer (UC). The solid lines indicate direct interaction whereas dashed lines correspond to indirect relationship among the interacting proteins, pathways and disease. The node shapes of the protein, molecular classes and relationship labels as outlined in the legends in the inserted boxes

Through IPA, we explored the interactions and pathways among *GNAQ*, *SEPTIN6*, *NTRK3*, and *IKZF1* (Fig. 4). *SEPTIN6* exhibited no connections in the protein network. *GNAQ*, *NTRK3*, and *IKZF1* were analyzed using the build tool to determine how their relationships are affected by UC. The protein network associated with the three main molecules is involved in the canonical signaling pathways of bladder cancer, PI3K/AKT, and mTOR. Each molecule was treated as a seed, and the network was constructed by connecting these seeds, thereby enabling us to explore the relationships among them. As indicated in Fig. 4, GNAQ, NTRK3, and IKZF1 may regulate UC through AKT1, MTOR, PI3KR1, HRAS, SRC, TP53, CCDN1, and RUNX3. The relationships among molecules involve activation, causation, correlation, expression, phosphorylation, protein–protein binding, were indicated in the network.

**Fig. 5:**
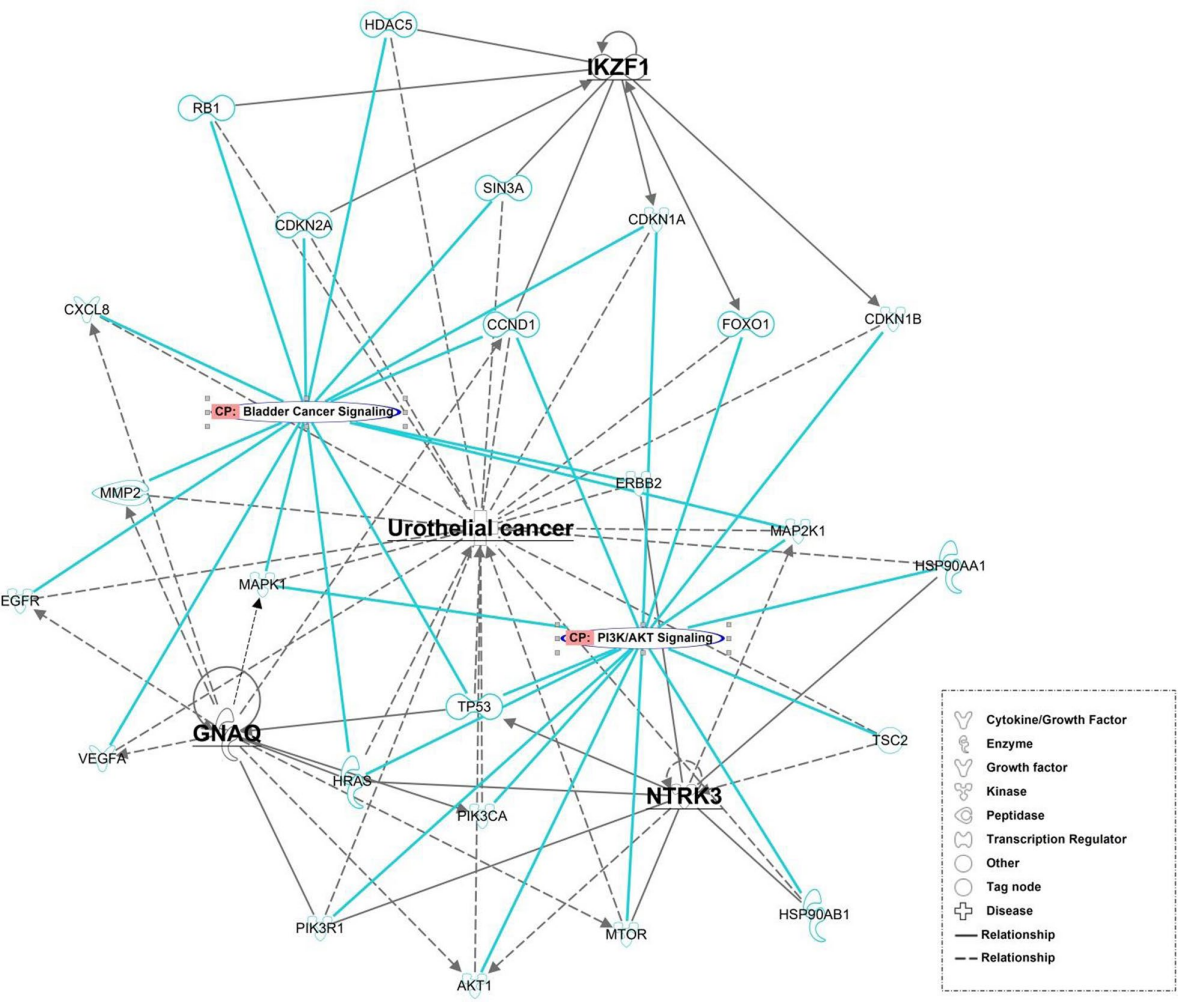
Protein network of Urothelial cancer (UC) relevant target protein with canonical pathway (CP). The molecules represented in CP were bladder cancer signaling and PI3K/AKT signaling. The relationships represented in light blue lines were genes involved in CP related to NTRK3, IKZF1, GNAQ, respectively

As indicated in Fig. 5, the network analysis revealed that GNAQ, NTRK3, and IKZF1 are related to UC because they are involved in PI3K/AKT and bladder cancer signaling. The 25 interacting proteins in the network (AKT1, CCND1, CDKN1A, CDKN1B, CDKN2A, CXCL8, EGFR, ERBB2, FOXO1, HDAC5, HRAS, HSP90AA1, HSP90AB1, MAP2K1, MAPK1, MMP2, MTOR, PIK3CA, PIK3R1, RB1, SIN3A, TP53, TSC2, VEGFA, and RUNX3) warranted further investigation. Thus, the result from IPA analysis explored the target proteins and networks that potentially contribute to UC and bladder cancer signaling pathways, which may inform future studies of the underlying mechanisms of UCKT.

## Discussion

Mutational profile analysis is the key to understanding the tumorigenic mechanisms of UC. The mutational profiles of UC that develop after transplantation have not been thoroughly investigated. In this study, WES analysis allowed us to characterize the mutational signatures of UCKT in detail. The comparison of CNV data in UCKT and UCHD revealed a subset of unique events and was warranted further investigation. Specific nucleotide conversion patterns that develop in certain cancers can elucidate the establishment of these mutations [45, 46]. In a TCGA study, 51% of all the mutations in bladder cancers were TpC > T or G mutations and 3.8% were TpC > A mutations [15]. In addition, *TP53, PIK3CA*, and *FGFR3* are the most common molecular alterations associated with UC [15, 47].

Tumors developed after kidney transplants are believed to be related to various factors, including immunosuppressive agents, viral infections, geographic area, and uremic environment, in patients with CKD/ESKD [48–50]. In the present study, missense mutations were the most common somatic mutations, followed by nonsense mutations. Furthermore, T > A replacements were the most common point mutations. Aristolochic acid (AA), which originates from plants in the genus *Aristolochia* that were used in herbal medicine, is a powerful nephrotoxin and human carcinogen associated with CKD and upper urinary tract UC (UUC) [51]. Aristolactam–DNA adducts, which are found in urothelial tissues [52], initiate mutation in the *TP53* tumor suppressor gene, creating a biomarker specific to AA-induced UUC [52, 53]. Taiwan has an extraordinarily high incidence of UUC due to the widespread use of *Aristolochia* herbal therapies [54], which suggests that AA might play a central role in the etiology of UUC. The mutation pattern discovered in our patients suggests that a possible history of exposure to *Aristolochia* remedies led them to develop ESKD and UC.

Four somatic mutations (*GNAQ, SEPTIN6, NTRK3*, and *IKZF1*) identified in current study may be related to UCKT. All these mutations were single-base substitutions specifically identified in the UCKT group, and none of the genes had been reported in the genomes of patients with UC. In our study, these mutations were detected in more than one UCKT genome, which suggests they did not occur randomly and potentially played roles in the pathogenesis of UCKT. According to the protein network in IPA, GNAQ, NTRK3, and IKZF1 may regulate UC through AKT1, MTOR, PI3KR1, HRAS, SRC, TP53, CCDN1, and RUNX3. Therefore, PI3K/AKT and bladder cancer signaling and their associated pathways must be further investigated in the future.

G proteins play an essential role in cellular signal transduction. The Gq protein alpha subunit (encoded by *GNAQ*) couples with a seven-transmembrane receptor to activate phospholipase C-beta, which generates second messengers, and to activate kinase cascades in the cytoplasm of the cells [55]. These signals ultimately control gene transcription and cell survival, motility, and growth. However, signals transmitted by G-protein-coupled receptors/G proteins and downstream targets are involved in the initiation and progression of cancer [56]. *GNAQ* is located on chromosome 9q21. The role of *GNAQ* as an oncogene has been observed in uveal melanoma [57], blue nevi [58], and malignancies affecting the meninges [59].

*NTRK3* is a member of the NTRK-encoding family, which comprises the genes *NTRK1*, *NTRK2*, and *NTRK3* that encode TrkA, TrkB, and TrkC, respectively [60]. *NTRK3* is located on chromosome 15q25. NTRK receptors signal through the JAK/STAT, PI3K/AKT, and MEK/ERK pathways to promote cell proliferation, differentiation, and survival [61]. Although numerous studies have investigated the key roles of these receptors in the development and function of the central and peripheral nervous system [62], alterations in *NTRK* genes have been observed in patients with colon [63], thyroid [64, 65], lung [66], glial [67], and breast [68] cancers. These alterations occur at relatively low frequencies (< 1%) among patients with each of these types of tumors, but *NTRK*-driven cancers affect numerous patients, making them key therapeutic targets [69–71]. *NTRK* gene fusions have been implicated in several diseases, and numerous therapeutic inhibitors have been developed [72]. Exploring the possible effect of *NTRK3* fusion on the development of UCKT among our cohort was worthwhile.

*IKZF1* encodes a transcription factor that belongs to the family of zinc finger DNA-binding proteins associated with chromatic remodeling. *IKZF1* is located on chromosome 7p12. *IKZF1* expression was highly conserved, which suggests its fundamental role in the ontogeny of the lymphopoietic system across species [73]. Ikaros genes are a major determinant of hematopoietic lineage, especially that of lymphocytes [74]. *IKZF1* deletions and mutations affect B-cell precursor acute lymphoblastic leukemia and contribute to its poor prognosis [75]. Constitutional and acquired genetic changes in Ikaros genes have been associated with human diseases, including lung, ovarian, liver, and colorectal cancers [74, 76–78].

Cell signaling pathways play key roles in tumorigenesis. Genetic and protein alterations in these pathways can modify cell cycle control, DNA repair, and carcinogen metabolism [79]. The mammalian target of rapamycin (mTOR) pathway is central to the development of multiple cancers, including UC [80]. mTOR is involved in complex signaling cascades that regulate cell growth and angiogenesis under both normal and cancerous conditions. Other key molecules involved in this pathway include upstream activators, such as PI3K; AKT; negative regulators, such as the tuberous sclerosis complex (TSC) 1/2; and downstream effectors, such as p70 S6 kinase and 4EBP1 [81]. The functional roles of our candidate genes (*GNAQ, NTRK3*, and *IKZF1)* in these pathways identified through IPA require further validation.

This study has several limitations. First, this was a retrospective study with a limited sample size. Second, due to limitations in sample collection, all the patients in our study groups were female; nonetheless, this is consistent with the finding that most UCKT individuals in the Taiwanese population are female [11, 82]. Additional studies with larger sample sizes are required to achieve conclusive results. Third, the contribution of the dbSNPs were not thoroughly discussed. Lastly, functional validation is necessary to identify the characteristics of each of the candidate genes.

## Conclusion

In conclusion, we identified 14 novel mutations of *CARD11*, *FNBP1*, *GNAQ*, *HOXD13*, *IKZF1*, *MAX*, *MLLT10*, *NTRK3*, *SEPTIN6*, *SEPTIN9*, *SH3GL1*, *SLC34A2*, *TAL1*, and *TRAF7* in a group of patients with UCKT. Among the affected genes, *GNAQ*, *IKZF1*, and *NTRK3* were potentially involved in the signaling network of UCKT. These findings could elucidate the development of UCKT and serve as a basis for the discovery of new potential biomarkers and the development of more effective treatments for UCKT.

## Supplementary Information

**Additional file 1: Fig. S1.** The comparison of SBS signature. Single-base substitution (SBS) signatures were analyzed by SigProfiler tool. The SBS96 Decomposition results of (A) UCKT, (B) UCHD and (C) COMIC UC were shown. The signatures and percentages of the SBS signatures were indicated, respectively.

**Additional file 2: Table S1.** Unique CNV in UCKT samples. CNV data was extracted from the WES results and divided into two groups. Total 1495 and 1033 events were observed in UCKT and UCHD groups. Using the start site as a reference, events occur in two or more patients in either group were selected. The records that were found in both groups were removed and only reported those that were specific in UCKT was retained.

## Data Availability

The data that support the findings presented in this manuscript are available from the corresponding author upon request.

## Abbreviations

ESKD: End-stage kidney disease
PTMs: Post-transplant malignancies
KT: Kidney transplantation
UC: Urothelial carcinoma
TCGA: The Cancer Genome Atlas
UCKT: Urothelial carcinoma post kidney transplantation
WES: Whole-exome sequencing
UCHD: Urothelial carcinoma of hemodialysis patients
CKD: Chronic kidney disease
SNVs or SNPs: Single-nucleotide variants/polymorphisms
InDels: Insertions or deletions
FFPE: Formalin-fixed paraffin-embedded
DNA: Deoxyribonucleic acid
CCG: Cologne Center for Genomics
BWA: Burrows-wheeler Alignment
SBS: Single-base substitution
GATK: Genomic Analysis Toolkit
COSMIC: Catalogue of Somatic Mutations in Cancer
IntOGen: The Integrative Onco Genomic
PCR: Polymerase chain reaction
PBMC: Peripheral blood mononuclear cell
IPA: Ingenuity Pathway Analysis
GNAQ: Guanine nucleotide-binding protein G(q)
CNV: Copy number variations
NTRK3: Neurotrophic tyrosine receptor kinase 3
IKZF1: Ikaros family zinc finger protein 1
AA: Aristolochic acid
UUC: Upper urinary tract urothelial carcinoma
mTOR: Mammalian target of rapamycin
