# Supplementary figures and images for: Whole-exome sequencing identified mutational profiles of urothelial carcinoma post kidney transplantation
Source: J Transl Med. 2022 Jul 21;20:324. doi: 10.1186/s12967-022-03522-4 (PMC9301867; doi:10.1186/s12967-022-03522-4)

(A)

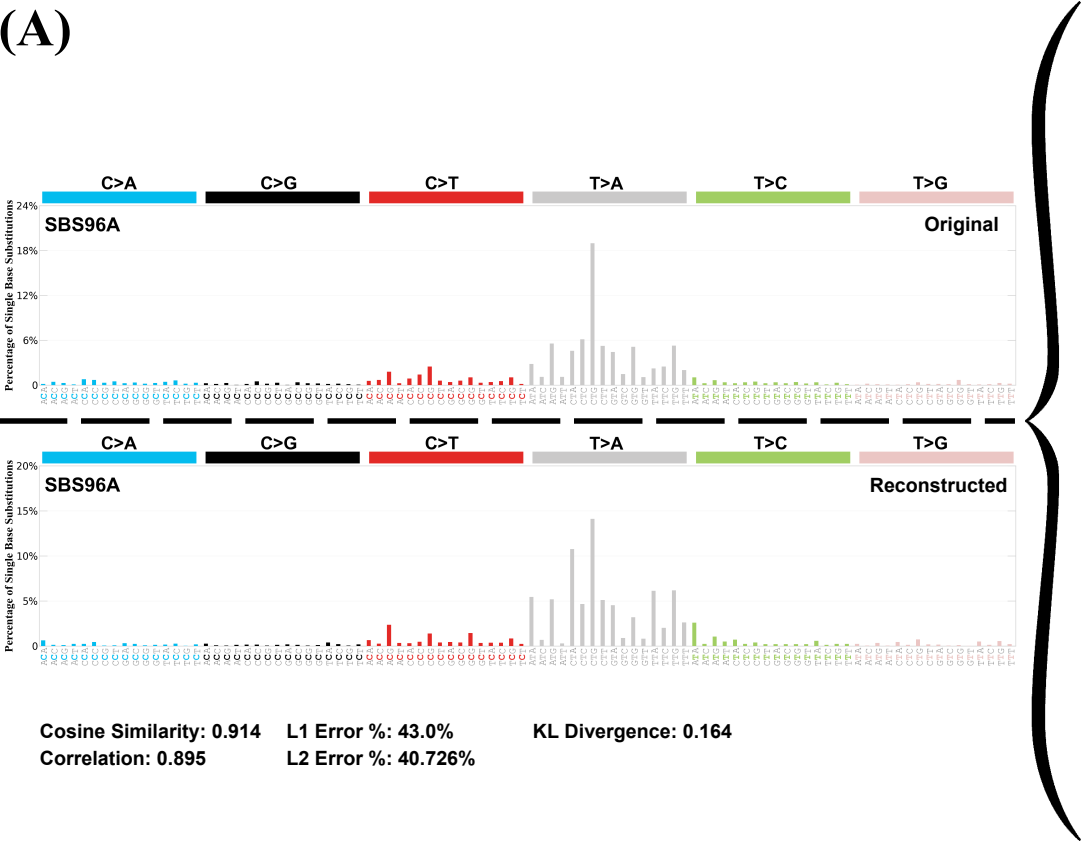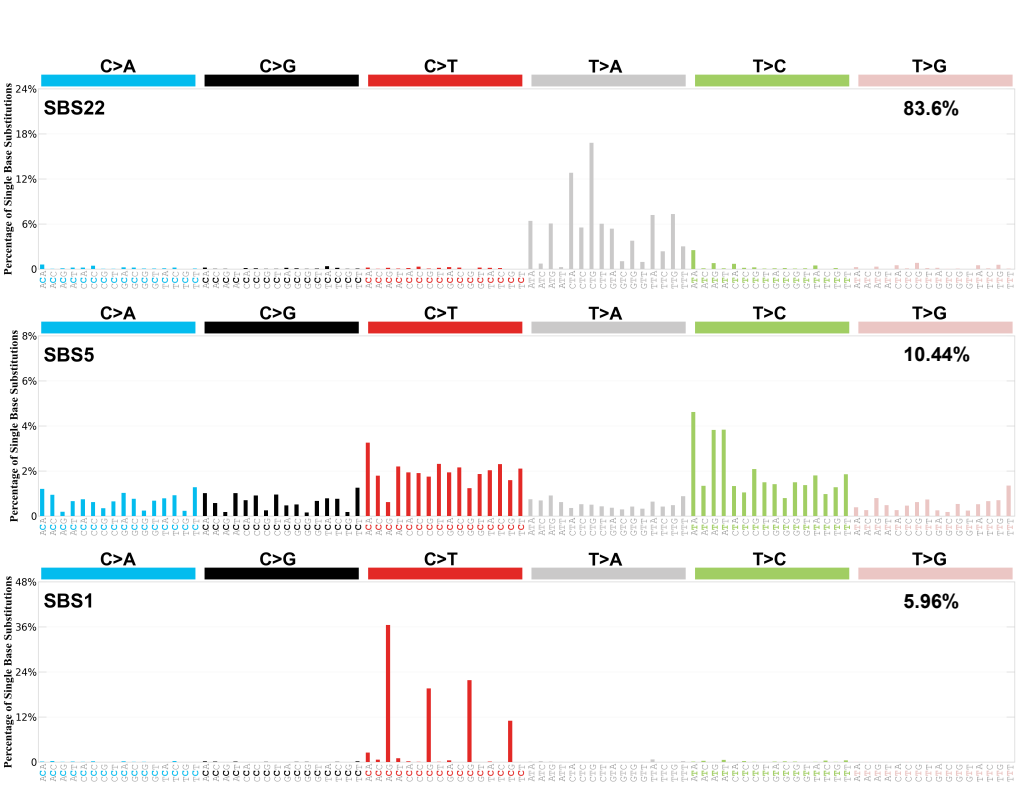

(B)

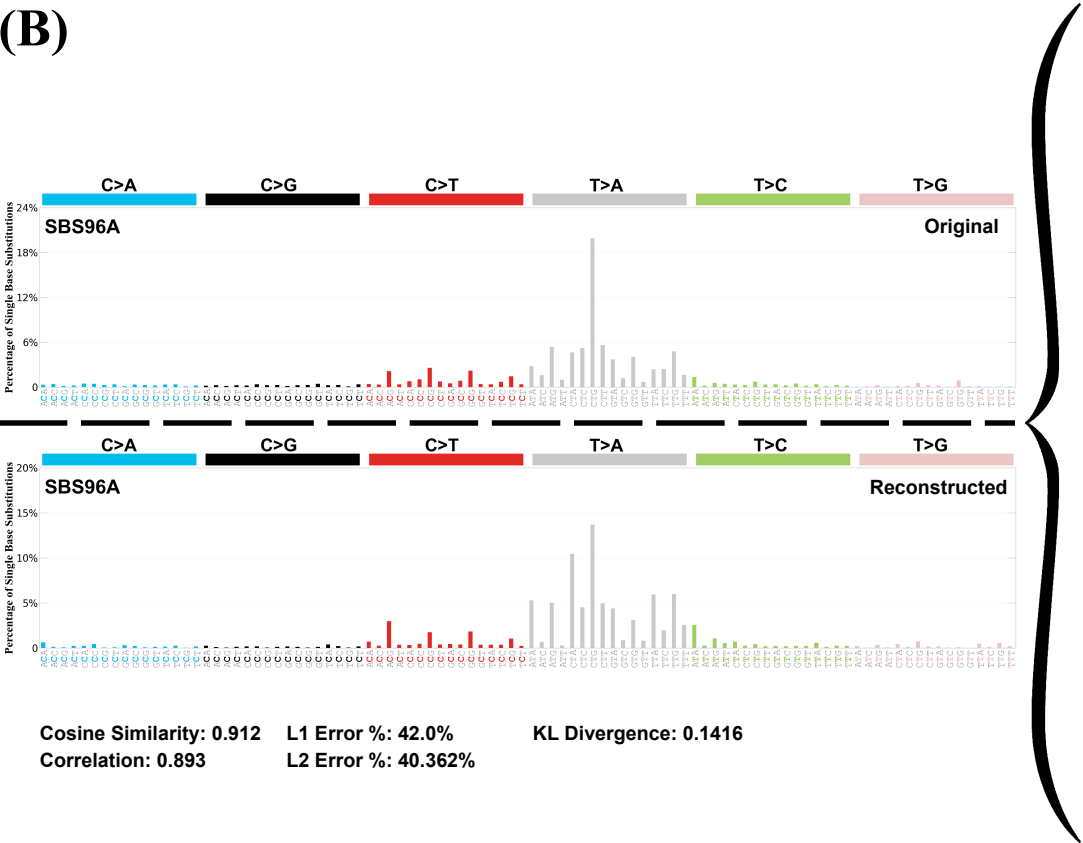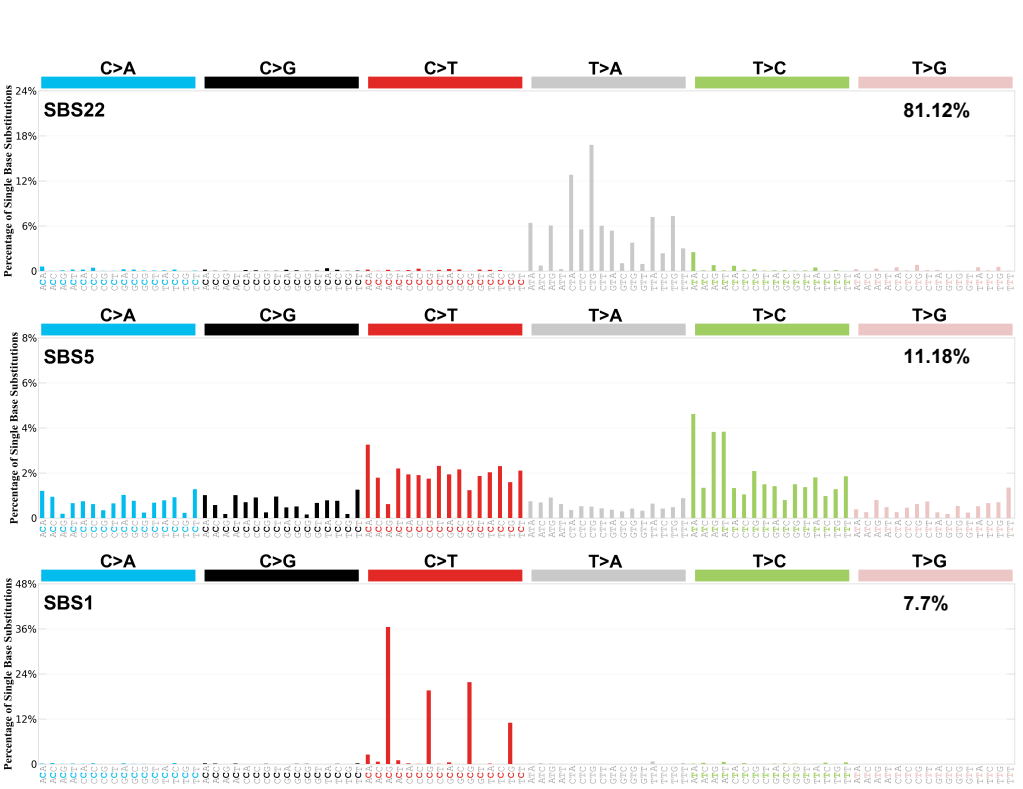

(C)

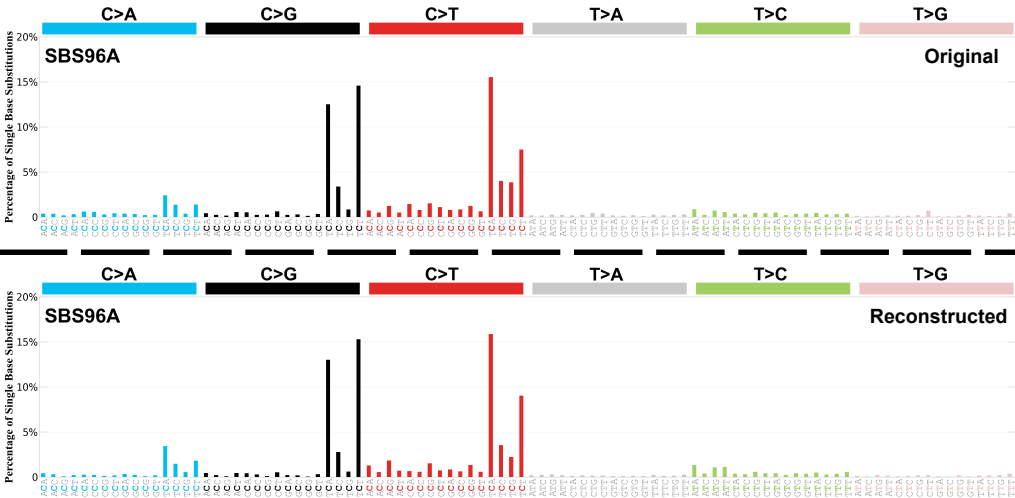

Cosine Similarity: 0.994    L1 Error %: 18.0%    KL Divergence: 0.0428  
Correlation: 0.993    L2 Error %: 11.037%

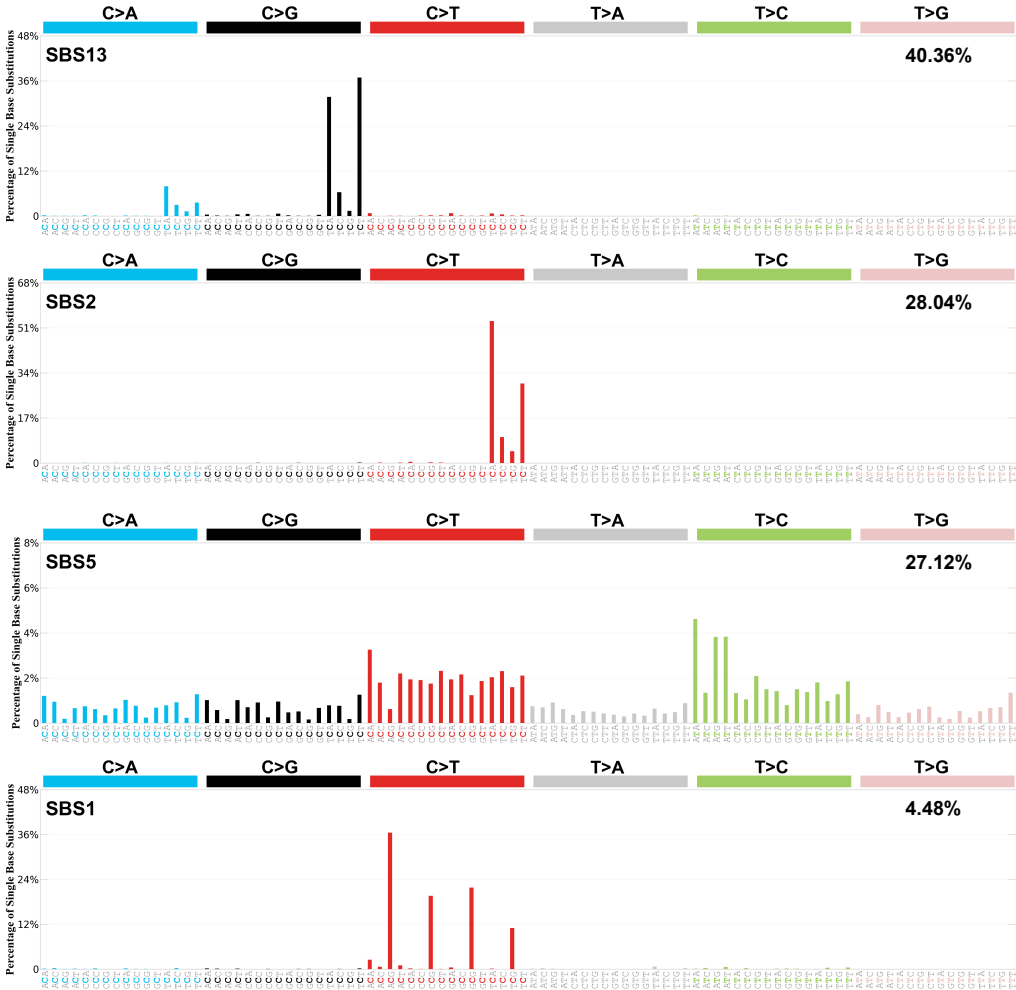

Supplement: Supplementary file 1 — Additional file 1: Fig. S1. The comparison of SBS signature. Single-base substitution (SBS) signatures were analyzed by SigProfiler tool. The SBS96 Decomposition results of (A) UCKT, (B) UCHD and (C) COMIC UC were shown. The signatures and percentages of the SBS signatures were indicated, respectively. [file 12967_2022_3522_MOESM1_ESM.pdf]
